# Follistatin-like protein 1: a serum biochemical marker reflecting the severity of joint damage in patients with osteoarthritis

**DOI:** 10.1186/ar3522

**Published:** 2011-11-25

**Authors:** Yuji Wang, Dawei Li, Nanwei Xu, Weijian Tao, Ruixia Zhu, Rongbin Sun, Weiwei Fan, Ping Zhang, Tianhua Dong, Long Yu

**Affiliations:** 1 Department of Orthopaedics, Changzhou No.2 People's Hospital, the Affiliated Hospital of Nanjing Medical University, 29 Xinglong Alley, Changzhou, 213003, P. R. China; 2 State Key Laboratory of Genetic Engineering, Institute of Genetics, School of Life Sciences, Fudan University, 220 Handan Road, Shanghai, P. R China; 3 Department of Orthopaedics, Suzhou Xiangcheng People's Hospital, 1060 Huayuan Road, Suzhou, 215131, P. R. China; 4 Clinical laboratory, Changzhou No.2 People's Hospital, the Affiliated Hospital of Nanjing Medical University, 29 Xinglong Alley, Changzhou, 213003, P. R. China; 5 Department of Orthopaedics, the First Affiliated Hospital of Suzhou University, 188 Shizi Street, Suzhou, 215006, P. R. China

**Keywords:** FSTL1, osteoarthritis, biochemical marker, KL grade, WOMAC score

## Abstract

**Introduction:**

Follistatin-like protein 1 (FSTL1) is a secreted glycoprotein that has been implicated in arthritis pathogenesis in a mouse model. The aim of this study is to detect FSTL1 expression and to further assess its potential utility as a biomarker of joint damage in osteoarthritis (OA) patients.

**Methods:**

FSTL1 expression was detected by real-time PCR, western blot and immunohistochemistry (IHC) in the synovial tissues (STs) and by IHC in the articular cartilage from OA patients and control trauma patients. The serum and synovial fluid (SF) FSTL1 concentrations were measured by ELISA in OA patients and control individuals. Linear regression analyses were used to assess correlations between the serum FSTL1 levels and the clinical characteristics in OA patients.

**Results:**

The FSTL1 mRNA and protein levels were substantially elevated in the STs from OA patients compared with those from control trauma patients. The FSTL1 expression was strong in the cytoplasm of the synovial and capillary endothelial cells of the STs, but weak in the chondrocytes of the articular cartilage from OA patients. Furthermore, the serum and SF FSTL1 concentrations were significantly higher in OA patients than in respective control subjects. Interestingly, the serum and SF FSTL1 levels were markedly higher in female OA patients than in males. Importantly, bivariate regression analysis revealed that the serum FSTL1 levels in female OA patients had significant correlations with Kellgren and Lawrence (KL) grade, joint space narrowing (JSN) and the Western Ontario McMaster and Universities Osteoarthritis (WOMAC) stiffness subscale, an inverse correlation with height, and marginal correlations with the total WOMAC score and the WOMAC function subscale. Multivariate regression analysis revealed that the serum FSTL1 levels correlated independently with KL grade in female OA patients. Bivariate analysis also revealed that the serum FSTL1 levels correlated significantly with age and disease duration, and they correlated marginally with high sensitivity C-reactive protein (hs-CRP) and KL grade in male OA patients.

**Conclusions:**

Increased FSTL1 expression may be a characteristic of OA patients. FSTL1 is a potential serum biomarker that may reflect the severity of joint damage, and further studies are required to evaluate its potential application for monitoring the course of the disease and the efficacy of therapies in OA patients.

## Introduction

Osteoarthritis (OA) is characterized by the destruction of articular cartilage and subchondral bone and by synovitis [1,2]. The progressive deterioration of joint structure and function has prompted studies to identify potential biomarkers of the damage to articular cartilage and subchondral bone as well as markers to monitor the progression of the disease and facilitate the design of proper treatment [3,4]. In addition, many of the biomarkers have also been implicated in OA pathogenesis, broadening our insight into this process.

FSTL1 is an extracellular glycoprotein originally cloned from a mouse osteoblast cell line as a transforming growth factor β (TGFβ)-inducible gene [5]. FSTL1 is widely expressed in human tissues and induced by ischemic stress and proinflammatory mediators [6-9]. Although the functions of FSTL1 at the molecular level remain largely unknown, several reports indicate that FSTL1 is involved in arthritis pathogenesis. Tanaka *et al*. [10] first identified FSTL1 as an autoantigen in systemic rheumatic diseases, and found FSTL1 and its autoantibody in synovial fluid (SF) and plasma in patients with rheumatoid arthritis (RA). Further studies demonstrate that FSTL1 has potential preventive effects on joint destruction by inhibiting the production of matrix metalloproteinases (MMPs) and cytokines both in synovial cells *in vitro *and in mouse models *in vivo *[11,12]. Consistently, one recent report reveals that FSTL1 plays an immunomodulatory role in heart allograft transplantation by inhibition of the proinflammatory cytokines, IL6, IL17A and IFNγ [13]. Another report indicates that FSTL1 protects the kidneys from acute nephrotoxic injury by inhibiting IL-1β production [14]. However, conflicting data have also shown that FSTL1 is a new proinflammation mediator that causes and aggravates arthritis by promoting the expression of IL-1β, TNFα and IL-6 and by enhancing the IFNγ signaling pathways in a mouse model [8,15].

Although the effects of FSTL1 on inflammation and immunity are complex and controversial, serum FSTL1 concentrations have been assessed in healthy individuals and patients with acute coronary syndrome (ACS), and shown to relate to ACS mortality during follow-up [16]. A recent study also reveals that serum FSTL1 levels are increased in systemic on-set juvenile rheumatoid arthritis (JRA) and suggests that FSTL1 may represent a biomarker of disease activity in systemic JRA [17]. Our previous report indicates that the serum FSTL1 levels are elevated in patients with RA, ulcerative colitis, systemic lupus erythematosus, Sjögren's syndrome, systemic sclerosis and polymyositis/dermatomyositis [9]. Furthermore, the serum FSTL1 levels correlate with inflammatory status and represent a new biomarker for disease activity in the RA population [9].

However, FSTL1 expression and its correlations with the clinical characteristics in OA patients have not been assessed until now. The present study is aimed at determining FSTL1 levels and further evaluating its clinical potential practicality as a serum biomarker reflecting the severity of joint damage in OA patients.

## Materials and methods

### Subjects

All OA and RA patients included in this study were diagnosed according to the American College of Rheumatology (ACR) clinical classification criteria [18-20]. The samples of synovial tissues (STs) from 33 OA patients and 10 RA patients were obtained by total knee joint replacement or arthroscopic synovectomy. These tissue samples were inspected visually to ensure that inflamed tissues with obvious edema and erythema were included. The control knee tissue samples were taken from seven trauma patients, including four patients with femur fractures and two patients with tibial and fibular fractures who underwent transarticular intramedullary interlocking nail insertion and one patient who underwent amputation due to severe trauma. These trauma patients had healthy joints with intact synovium. Most of the tissue samples were stored at -70°C immediately upon acquisition until use for mRNA and protein extraction. Some tissue samples were also fixed in 4% paraformaldehyde and embedded in paraffin. The SF samples were collected from 42 OA patients, 16 RA patients and 6 trauma patients who served as controls. The SF samples were collected by means of needle aspiration with the use of a sterile technique during knee joint surgery. The SF samples were placed in a sterile polypropylene tube and then frozen at -70°C until analysis. The mRNA sample from one trauma control was not acquired because of the relatively small tissue blocks. We were also unable to acquire the SF sample from another trauma patient. The clinical and demographic characteristics of these patients and of the controls whose SF samples were available are shown in Table 1.

**Table 1:** Characteristics and SF FSTL1 levels in the subjects investigated^a^

	Con	OA	RA	Male OA	Female OA
Number	6	42	16	11	31
Age (years)^b^	32 (30-49)	67 (60-72)	61 (54-70)	70 (55-75)	67 (60-72)
Sex (M/F)	4/2	11/31	2/14	-	-
Disease duration (years)^b^	-	1.0 (0.5-3.0)	2.0 (0.5-4.5)	0.9 (0.2-2.8)	1.0 (0.5-6.0)
FSTL1 levels (μg/L)^b^	6.80	15.66	39.80	11.65	16.54
	(6.02-7.32)	(12.04-19.80)	(22.74-273.80)	(8.57-13.50)	(14.09-27.53)

^a^Included all subjects for whom SF samples were available and part of subjects for whom tissue samples were available. ^b^Expressed as the median (25^th ^to 75th percentile). Con, control trauma patients; FSTL1, follistatin-like protein 1; OA, osteoarthritis; RA, rheumatoid arthritis.

The serum samples were collected by venipuncture from 168 OA patients including 158 knee and 10 hip OA patients. The clinical data were carefully reviewed to exclude any other forms of inflammatory joint diseases, systemic autoimmune diseases, as well as tumors or heart, liver or kidney diseases. The OA patients included untreated individuals (88%), patients treated with nonsteroidal anti-inflammatory drugs (NSAIDs) (10%) or patients treated with joint space injection of sodium hyaluronate (2%). A total of 63 apparently healthy control individuals (HCs) who were without regular medication or chronic drug use or other chronic diseases or acute illnesses were included. The serum samples were acquired and stored immediately at -20°C until measurement. The demographic, clinical and laboratory characteristics of the subjects for whom the serum samples were available are summarized in Table 2. In this study, 32 OA patients (8 male and 24 female) had the paired serum and SF samples; 13 OA patients had the paired serum and ST samples; 17 OA patients had the paired ST and SF samples. A total of 10 OA patients had the matched serum, SF and ST samples.

**Table 2:** Characteristics and serum FSTL1 levels in the subjects investigated^a^.

	HC	OA	Male HC	Male OA	Female HC	Female OA
Number	63	168	29	48	34	120
Age (years)^b^	37 (29-47)	62 (54-69)	35 (31-43)	61 (54-72)	39 (29-52)	62 (54-69)
Disease duration (years)^b^	-	1.0 (0.3-3.9)	-	0.5 (0.2-3.0)	-	1.0 (0.5-5.0)
FSTL1 levels (μg/L)^b^	2.93	19.03	2.50	5.64	3.32	24.40
	(2.35-3.69)	(7.41-27.98)	(2.17-2.96)	(3.81-8.75)	(2.43-8.67)	(15.65-31.17)

^a^Included all subjects for whom serum samples were available. ^b^Expressed as the median (25^th ^to 75th percentile). FSTL1: follistatin-like protein 1; HC: healthy control individual; OA: osteoarthritis.

The erythrocyte sedimentation rate (ESR) was measured by the Westergren method. The serum high sensitivity C-reactive protein (hs-CRP) and rheumatoid factor (RF) levels were determined in a clinical laboratory using a latex photometric immunoassay. A 100-mm visual analogue scale (VAS) was used to calculate patients' baseline pain score. The Western Ontario McMaster and Universities Osteoarthritis (WOMAC) Index Likert Format 3.1 was used to assess the patients' physical function [21].

The OA severity was determined using weight-bearing anteroposterior radiographs of the affected joints, which were evaluated according to the Kellgren and Lawrence (KL) classification [22]. The grade of OA was described as follows: Grade 0, no radiographic findings of osteoarthritis; Grade 1, minute osteophytes of doubtful clinical significance; Grade 2, definite osteophytes with unimpaired joint space; Grade 3, definite osteophytes with moderate joint space narrowing (JSN); Grade 4, definite osteophytes with severe JSN and subchondral sclerosis. Each radiograph was examined independently by two investigators (YW and TD) in a blinded manner. The inter-rater reliability and intra-rater reliability for the investigators were high (the weighted kappa for inter-rater reliability was 0.93; the kappa for intra-rater reliability was 0.94). Nine inconsistent radiographs were arbitrated by another investigator (NX). Only the patients for whom serum samples were available had complete KL and partial WOMAC scores. Because we mostly used banked samples of ST and SF, we did not obtain KL and WOMAC scores from these patients, except in the cases of those who had paired serum samples.

Collection of the ST, SF and serum samples was performed according to the medical ethics regulations of Nanjing Medical University. This study was approved by Nanjing Medical University Review Board, and written permissions were requested and received from all patients and HCs.

### Real-time PCR

Total RNA extraction, retrotranscription and real-time PCR were carried out as previously described with minor modifications [9]. Briefly, total RNA was extracted from the minced cryogenic tissues using miRNeasy Mini kit (Qiagen, Basel, Switzerland) according to the manufacturer's instructions. In-column digestion of contaminating DNA was performed using the RNase-Free DNase Set (Qiagen). Total RNA (1 μg) was reverse-transcribed using the ReverTra Ace kit (Toyobo, Osaka, Japan) according to the manufacturer's instructions. Real-time PCR was performed using SYBR Green (Toyobo) in a Roche LightCycler^® ^480 (Roche Applied Science, Indianapolis, IN, US). The primer sequences for amplifying FSTL1 cDNA and internal control GAPDH were as follows: FSTL1, 5' -CGATGGACACTGCAAAGAGA-3' (forward) and 5'-CCAGCCATCTGGAATGATCT-3' (reverse); GAPDH, 5'-AGGGCTGCTTTTAACTCTGGT-3' (forward) and 5'-CCCCACTTGATTTTGGAGGGA-3' (reverse). The comparative threshold cycle (Ct) method was used for relative quantification of mRNA.

### Western blot

The minced cryogenic tissues were lysed and boiled in Laemmli buffer (50 mmol/L Tris-HCl pH 6.8, 2% SDS, 0.1% bromophenol blue, 10% glycerol, 5% β-mercaptoethanol) SDS-PAGE was performed on 12% polyacrylamide, and the proteins transferred to nitrocellulose membranes. A goat anti-human FSTL1 polyclonal antibody (R&D Systems, Minneapolis, Minnesota, US) and actin AC-40 (Sigma-Aldrich, St. Louis, MO, US) were used to detect FSTL1 and β-actin expression, respectively.

### Immunohistochemistry

The samples of STs from four control trauma patients and four OA patients, as well as cartilage tissues from one control trauma patient and one OA patient, were assessed by immunohistochemistry (IHC). IHC staining was performed on archival formalin fixed, paraffin-embedded tissues, using the peroxidase technique as described previously [9]. Briefly, sections were deparaffinized and rehydrated. Epitope retrieval was performed in citrate buffer (pH 6) in a water bath at 95°C for 15 minutes with cooling for 10 minutes before immunostaining. The tissues were incubated with the anti-FSTL1 antibody (1:400 dilution) overnight at 4°C after blocking and then exposed to a biotinylated secondary linking antibody (Boster, Wuhan, China) for 20 minutes. Biotin detection was performed with peroxidase-conjugated streptavidin (Boster). Finally, the slides were incubated with a 3, 3'-diaminobenzidine (DAB) solution (Boster) for 1 to 5 minutes and counterstained with hematoxylin for 1 minute. The sections were washed between incubations with Tris-buffered saline (pH 7.6). A goat polyclonal immunoglobulin G (IgG) was used as a negative control throughout the procedure. Each section was examined independently by two investigators (DL and YW) in a blinded manner.

### Enzyme-linked immunosorbent assay (ELISA)

The serum and SF FSTL1 concentrations were detected using a standard quantitative sandwich ELISA (Groundwork Biotechnology Diagnosticate, San Diego, CA, US) with a detection limit of 100 pg/ml. The SF samples were pretreated with 5 mg/ml hyaluronidase (Sigma-Aldrich, St. Louis, MO, US) dissolved in phosphate buffered saline for 5 minutes and clarified by means of centrifugation at 5,000 g for 10 minutes. Aliquots of 50 μl of the collected SF supernatant or serum samples were added to microwells. A total of 100 μl of the conjugated antibody was added to each microwell and mixed thoroughly with the samples. After extensive washing, a substrate solution was added that reacts with enzymes to produce a color change. The optical densities were determined using a microplate reader Elx808™ (Bio-Tek Instruments, Winooski, VT, USA).at 450 nm. Software Gene 5 (BioTek Instruments) was used to analyze all materials and produce a standard curve. The concentrations were reported as μg/L. All analyses and calibrations were performed in duplicate.

### Statistical analysis

Statistical analyses were performed using Prism and Instat (GraphPad Software, San Diego, CA, US) and SPSS 13.0 software (SPSS Inc., Chicago, IL, US). The significance of the differences was evaluated using the Kruskal-Wallis test among multiple groups and using the Mann-Whitney U test between groups. The paired serum and SF samples were evaluated by the Wilcoxon matched pairs test. Bivariate linear regression analysis was used to evaluate the relationships between serum FSTL1 levels and other parameters. Multivariate linear regression analysis was performed to identify variables that were independently associated with serum FSTL1 levels. Variables that were not normally distributed were transformed to their natural logarithm (ln) for the regression analyses. The Kolmogorov-Smirnov test was used to assess the normal distribution of variables. For regression analysis, the binary variables were coded as follows: hypertension (0 = no hypertension, 1 = hypertension), diabetes (0 = no diabetes, 1 = diabetes), bilateral joints involved (0 = single joint involved, 1 = bilateral joints involved) and JSN (0 = no JSN, 1 = JSN). All quoted *P *values were 2-tailed, and *P *values less than 0.05 were considered significant.

## Results

### Increased FSTL1 expression in the STs from OA patients

**Figure 1:**
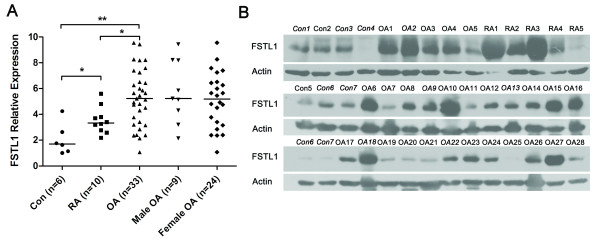
**Increased FSTL1 expression in the STs from OA patients**. **(A) **FSTL1 mRNA expression in the cryogenic tissue samples from control trauma patients (Con), OA and RA patients, male OA patients and female OA patients. The results are expressed in relative units. The horizontal lines represent the medians. Each point represents an individual value. *, *P *< 0.05; **, *P *< 0.01. **(B) **FSTL1 protein expression in the cryogenic tissue samples from control trauma patients (Con1 to Con7: normal STs from 7 different trauma controls), RA patients (RA1 to RA5: RA STs from 5 different RA patients) and OA patients (OA1 to OA28: OA STs from 28 different OA patients). The male individuals are represented in italics. The FSTL1 immunoreactive band appears as a band with an electrophoretic mobility corresponding to 46 kDa. β-actin was used as a loading control. FSTL1, follistatin-like protein 1; Con, control trauma patients; RA, rheumatoid arthritis; OA, osteoarthritis; ST, synovial tissue.

To assess the FSTL1 expression levels in OA patients, we first compared FSTL1 mRNA levels in the STs of OA, RA and control trauma patients. Consistent with our previous report [9], the FSTL1 mRNA levels were upregulated in the STs of RA patients by approximately 1.71-fold compared with the controls. Interestingly, we also found that the FSTL1 mRNA levels in OA patients were elevated by approximately 1.49-fold and 2.55-fold compared with RA patients and the controls, respectively (Figure 1A). However, the FSTL1 mRNA levels showed no difference between male and female OA patients. Furthermore, we detected FSTL1 protein expression by western blot in the representative tissue samples. The FSTL1 protein levels were markedly higher in the STs from OA and RA patients than in the controls (Figure 1B). These results indicate that upregulation of FSTL1 expression generally occurs in the STs of OA and RA patients.

### Localization of FSTL1 in the STs from OA patients

**Figure 2:**
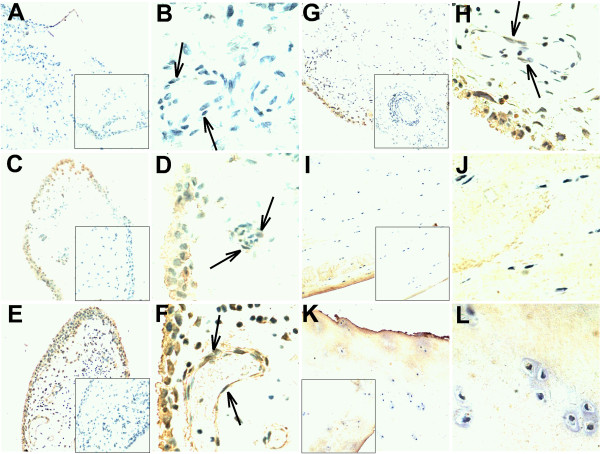
**Immunohistochemical staining of FSTL1 in the STs and articular cartilage from control trauma patients and OA patients**. **(A-B) **The STs from control trauma patients. The synovial cells of the lining and sublining with little or weak FSTL1 immunostaining are shown. **(C-H) **The STs from OA patients. The synovial cells of the lining and sublining with positive FSTL1 immunostaining are shown. **(I-L) **The articular cartilage from control trauma patients (I-J) and OA patients (K-L). The chondrocytes with little or weak FSTL1 immunostaining are shown. The STs (C and D; E and F; G and H) were collected from three different OA patients. The STs (G and H) and articular cartilage (K and L) were collected from the same OA patients. All tissue samples were counterstained with hematoxylin. (A, C, E, G, I and K) Original magnification, ×100. (B, D, F, H, J and L) Original magnification, ×400. The negative controls stained with polyclonal goat IgG (original magnification, ×100) are shown in the insets (A, C, E, G, I and K). Arrows indicate the capillary endothelial cells in the STs from control trauma patients (B) and OA patients (D, F and H). Representative images from one of three separate experiments with similar results are shown. FSTL1, follistatin-like protein 1; IgG, immunoglobulin G; OA, osteoarthritis; ST, synovial tissue.

To further assess the FSTL1 expression levels and tissue distribution, we stained the sections of the STs from both OA patients and control trauma patients using a specific antibody for FSTL1 (Figure 2A-2H). The FSTL1 staining was at a relatively low level in the normal STs from the controls (Figure 2A-2B), but was much more intense in the STs from OA patients compared with the controls (Figure 2A-2H). The FSTL1 staining was clearly seen in the cytoplasm of the synoviocytes in the lining and sublining from the STs of OA patients (Figure 2C-2H). Interestingly, the FSTL1 expression was also stronger in the endothelial cells (ECs) of the OA samples than in the controls (Figure 2B, D, E and 2H), suggesting a possible role in angiogenesis in arthritis. In contrast, the FSTL1 staining was invisible in most of the infiltrated lymphocytes. Finally, we assessed FSTL1 expression on the sections of the articular cartilage from OA patients and the controls (Figure 2I-2L). The FSTL1 staining was weak or even absent in the chondrocytes of the articular cartilage, and showed no differences between OA patients and the controls (Figure 2I-2L). Interestingly, the FSTL1 expression was markedly stronger in the synovial cells and ECs than in the chondrocytes from the same OA patient (Figure 2G-2H and Figure 2K-2L).

### Elevated serum and SF FSTL1 levels in OA patients

**Figure 3:**
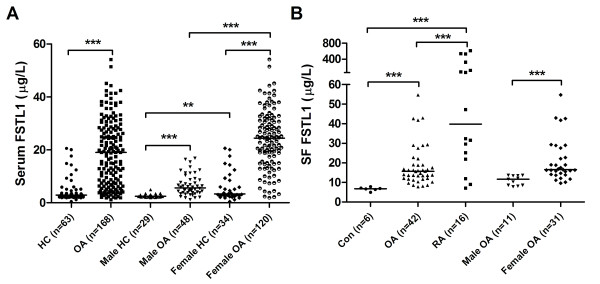
**Elevated serum and SF FSTL1 levels in OA patients**. **(A) **Serum FSTL1 concentrations in HCs, OA patients, male HCs, male OA patients, female HCs and female OA patients. **(B) **SF FSTL1 concentrations in control trauma patients (Con), OA and RA patients, male OA patients and female OA patients. The horizontal lines represent the median values. Each point represents an individual value. Con, control trauma patients; FSTL1, follistatin-like protein 1; HC, healthy control individual; OA, osteoarthritis; RA, rheumatoid arthritis; SF, synovial fluid. **, *P *< 0.01; ***, *P *< 0.001.

Next, we examined the serum FSTL1 levels in HCs and OA patients. The serum FSTL1 levels were much higher in OA patients than in HCs (Figure 3A and Table 2). Furthermore, we stratified all individuals by sex and compared the serum FSTL1 levels. We found that the serum FSTL1 levels were significantly elevated in female individuals compared to male individuals among both the HCs and the OA patients (Figure 3A and Table 2). Then, we selected age-matched HCs and compared the serum FSTL1 levels between the HCs and OA patients in male and female groups. The serum FSTL1 levels were also significantly higher in OA patients than in the HCs in both groups (data not shown). The serum FSTL1 distribution was heterogeneous between male and female OA patients (Additional file 1, Figure 1S). We also compared the SF FSTL1 levels among OA, RA and control trauma patients. The SF samples were pretreated with hyaluronidase to reduce their viscosity. The SF FSTL1 levels were increased significantly after treatment or following several cycles of freezing and thawing (data not shown), suggesting that FSTL1 could be tethered in a large molecule complex or released from viscous contaminants. The FSTL1 levels in the SF samples of the OA patients were significantly higher than those in the controls but lower than those in the RA patients (Figure 3B and Table 1). The SF FSTL1 levels were also significantly higher in the female OA patients compared to the males (Figure 3B and Table 1). Moreover, we compared the serum FSTL1 levels with the SF FSTL1 levels in 32 paired samples from the same OA patients and did not observe significant differences in both the male and female OA groups (data not shown).

### Serum FSTL1 levels correlate with KL grade in OA patients

Finally, we assessed the relationship between the serum FSTL1 levels and the demographic, clinical and laboratory characteristics in OA patients. We separately evaluated the relationship in male and female OA patients because serum FSTL1 distribution was obviously heterogeneous between the sexes (Additional file 1, Figure 1S).

The serum FSTL1 levels were distributed normally in 120 female OA patients. Bivariate regression analysis revealed that the serum FSTL1 levels had significant correlations with KL grade and JSN, but an inverse correlation with height. Moreover, the serum FSTL1 levels correlated significantly with the WOMAC stiffness subscale, and they correlated marginally with the total WOMAC score and WOMAC function subscale. However, the serum FSTL1 levels did not correlate with the other variables included (Table 3). Next, we focused on 112 female patients with OA of the knee by excluding 8 female patients with OA of hip and obtained similar results (Additional file 1, Table 1S). In a multivariate regression analysis that used serum FSTL1 concentrations as the dependent variable and included age, body weight, height, disease duration, bilateral joints involved, hypertension, diabetes, hs-CRP, RF and KL grade as the independent variables, only KL grade was independently associated with the serum FSTL1 levels (Table 4). The total r^2 ^value of this model was 0.24.

**Table 3:** Baseline characteristics of female OA patients according to serum FSTL1 levels.

	**Female serum FSTL1 levels (μg/L)**					
			
	**First quartile**	**Second quartile**	**Third quartile**	**Fourth quartile**	***P***	**r**
			
	(1.94-15.65)	(15.65-24.35)	(24.45-31.08)	(31.20-54.20)		
Number	30	30	30	30		
Age (years)	62 (52-67)	66 (58-72)	62 (56-70)	61 (54-65)	0.592	0.049
Disease duration (years)	1 (0.5-3.0)	1 (0.2-6.3)	1.5 (0.5-3.6)	2 (0.5-8.5)	0.282	0.099
Height, cm	160 (155-164)	158 (156-162)	158 (158-162)	158 (155-162)	0.049	-0.181
Weight, kg	65 (60-70)	62 (55-70)	65 (60-70)	60 (55-65)	0.272	-0.101
BMI	25.3 (22.9-27.1)	25.1 (22.0-28.1)	25.8 (24.3-27.5)	23.9 (22.6-26.1)	0.667	-0.040
Single/dual knee (hip)	22/8	21/9	21/9	22/8	0.795	0.024
Hypertension	10 (33%)	9 (30%)	12 (40%)	6 (20%)	0.309	-0.094
Diabetes	5 (17%)	2 (7%)	6 (20%)	2 (7%)	0.894	-0.012
Hs-CRP (mg/L)	1.4 (0.6-4.1)	2.2 (1.1-4.7)	1.9 (1.0-4.6)	1.9 (0.9-11.3)	0.113	0.146
ESR^a^	13 (6-26)	11 (8-20)	10 (7-20)	11 (5-20)	0.270	-0.114
RF	6.9 (2.7-12.9)	8.6 (2.8-14.6)	7.3 (3.1-12.7)	7.7 (3.5-14.6)	0.910	0.010
Pain (VAS mm)^b^	60 (50-70)	60 (50-78)	55 (40-78)	50 (36-70)	0.166	-0.143
WOMAC score (all normalized/100)^c^						
Pain subscale	57 (50-61)	57 (48-70)	50 (46-64)	61 (53-71)	0.129	0.177
Stiffness subscale	47 (42-56)	50 (38-61)	58 (42-71)	64 (52-73)	0.015	0.279
Function subscale	56 (52-68)	59 (50-68)	63 (48-77)	73 (58-90)	0.079	0.204
Total Score	56 (48-63)	56 (48-66)	61 (46-73)	68 (56-78)	0.051	0.214
KL grade (0-4)	6/7/13/1/3	0/5/8/7/10	0/4/10/10/6	0/6/2/11/11	<0.0001	0.406
JSN	4 (13%)	17 (57%)	16 (53%)	22 (73%)	<0.0001	0.419

Data are number (%) or median (25^th ^to 75th percentile). Linear regression was used to evaluate the relationships between FSTL1 levels and baseline characteristics and to calculate *P *values and the correlation coefficient, r. (Disease duration, hs-CRP, ESR and RF in the female OA patients were not normally distributed and were entered as ln-transformed variables). ^a^Data from ESR were available for 95 patients; ^b^Data from Pain VAS were available for 95 patients; ^c^Data from the total WOMAC score were available for 84 patients, and the pain subscale, stiffness subscale and function subscale were available for 75 patients, respectively. BMI, body mass index; ESR, erythrocyte sedimentation rate; FSTL1, follistatin-like protein 1; Hs-CRP, high sensitivity C-reactive protein; JSN, joint space narrowing; KL, Kellgren and Lawrence; OA, osteoarthritis; RF, rheumatoid factor; VAS, visual analogue scale; WOMAC, Western Ontario and McMaster Universities.

**Table 4:** Independent association of serum FSTL1 levels with baseline characteristics in 120 female OA patients^a^.

Characteristics	B	*P *value
Age, years	-0.215	0.051
Body weight, kg	-0.148	0.207
Height, cm	-0.165	0.471
Disease duration, years	0.023	0.972
Bilateral joints involved	-2.633	0.254
Hypertension	-2.386	0.263
Diabetes	2.294	0.447
Hs-CRP, mg/l	1.151	0.142
RF, IU/ml	0.192	0.801
KL grade	4.458	<0.0001

^a^Multivariate linear regression analysis was used to assess variables associated with serum FSTL1 concentrations. B, unstandardized B coefficient; Hs-CRP, high sensitivity C-reactive protein; IU, international units; KL, Kellgren and Lawrence; OA, osteoarthritis; RF, rheumatoid factor.

The serum FSTL1 levels correlated significantly with age, disease duration and RF, and they correlated marginally with hs-CRP and KL grade in 48 male OA patients by bivariate analysis (Table 5). The serum FSTL1 levels were not correlated with the other variables included. Furthermore, we focused on 46 male patients with OA of the knee by excluding 2 male patients with OA of the hip and obtained similar results (Additional file 1, Table 2S). No variable was independently associated with the serum FSTL1 levels in the multivariate models among the male patients.

**Table 5:** Baseline characteristics of male OA patients according to serum FSTL1 levels.

	**Male serum FSTL1 levels (μg/L)**					
			
	**First quartile**	**Second quartile**	**Third quartile**	**Fourth quartile**	***P***	**r**
			
	(1.19-3.81)	(3.81-5.40)	(5.88-8.62)	(8.79-16.88)		
Number	12	12	12	12		
Age (years)	58 (47-61)	60 (54-74)	58 (52-72)	69 (65-79)	0.005	0.396
Disease duration (years)	0.1 (0.1-1.7)	0.6 (0.2-4.8)	1.5 (0.5-3.8)	2.5 (0.2-6.8)	0.004	0.405
Height, cm	170 (166-174)	168 (166-170)	166 (162-170)	169 (168-170)	0.378	-0.130
Weight, kg	72 (66-76)	68 (64-76)	67 (60-75)	69 (61-77)	0.376	-0.131
BMI	24.2 (22.8-26.2)	24.6 (22.7-26.3)	24.0 (21.6-25.8)	24.0 (21.4-26.4)	0.511	-0.097
Single/dual knee (hip)	11/1	12/0	9/3	9/3	0.130	0.222
Hypertension	4 (33%)	5 (42%)	5 (42%)	6 (50%)	0.486	0.103
Diabetes	2 (17%)	1 (8%)	1 (8%)	3 (25%)	0.687	0.060
Hs-CRP (mg/L)	1.4 (0.7-1.9)	1.1 (0.4-10.7)	1.4 (0.6-2.9)	2.4 (0.7-15.5)	0.056	0.278
ESR^a^	8 (3-10)	4 (3-13)	5 (5-20)	12 (2-27)	0.957	-0.009
RF	3.8 (0.3-10.6)	7.2 (4.8-18.7)	11.1 (6.0-18.8)	9.0 (7.4-16.3)	0.021	0.332
Pain (VAS mm)^b^	55 (40-60)	50 (20-70)	50 (35-80)	50 (40-80)	0.371	0.145
WOMAC score (all normalized/100)^c^						
Pain subscale	64 (57-68)	64 (64-71)	60 (51-68)	59 (52-61)	0.407	-0.152
Stiffness subscale	58 (46-75)	61 (45-81)	49 (45-79)	54 (43-58)	0.636	-0.087
Function subscale	65 (54-83)	77 (46-90)	69 (35-90)	60 (52-65)	0.617	-0.092
Total Score	63 (55-85)	69 (51-80)	63 (44-78)	59 (52-63)	0.342	-0.168
KL grade (0-4)	5/4/2/0/2	4/2/3/1/2	3/1/5/1/2	2/2/4/1/3	0.069	0.265
JSN	2 (17%)	3 (25%)	3 (25%)	4 (33%)	0.127	0.224

Data are number (%) or median (25^th ^to 75th percentile). Linear regression was used to evaluate the relationships between FSTL1 and baseline characteristics and to calculate *P *values and the correlation coefficient, r. (Serum FSTL1 levels, disease duration, hs-CRP, ESR and RF in the male OA patients were not normally distributed and were entered as ln-transformed variables). ^a^Data from ESR were available for 39 patients; ^b^Data for Pain VAS were available for 40 patients; ^c^Data from the total WOMAC score were available for 34 patients, and the pain subscale, stiffness subscale and function subscale were available for 32 patients, respectively. BMI, body mass index; ESR, erythrocyte sedimentation rate; FSTL1, follistatin-like protein 1; Hs-CRP, high sensitivity C-reactive protein; JSN, joint space narrowing; KL, Kellgren and Lawrence; OA, osteoarthritis; RF, rheumatoid factor; VAS, visual analogue scale; WOMAC, Western Ontario and McMaster Universities.

## Discussion

In this study, we found that the FSTL1 expression levels were increased in the ST, SF and serum samples from OA patients. The serum FSTL1 levels correlated with KL grade and JSN in the female OA patients, and they also correlated marginally with KL grade in the males. These findings suggest that FSTL1 may represent a new potential serum biochemical marker reflecting the severity of joint damage in OA patients.

The FSTL1 mRNA expression levels were elevated in the STs of OA patients compared with RA patients and control trauma patients. However, the serum and SF FSTL1 levels were highest in RA patients among three groups, including those described in our previous study [9]. These discrepancies may be due to the fact that the STs of RA patients have a higher density of proliferative synovial cells, which could secrete much more FSTL1 into the serum and SF under the conditions of the proinflammatory microenvironment, although they have a relatively lower mean cellular FSTL1 mRNA expression.

The serum and SF FSTL1 levels were markedly increased in OA patients. Long-term chronic inflammation may stimulate synovial cell hyperplasia and neovascularization in the OA patients [23,24]. The large areas of synovitis with proliferative synovial cells and the increased vascular bed could induce more FSTL1 production in the OA patients. We also found that the female OA patients had higher serum and SF FSTL1 levels than the male OA patients. It is possible that female OA patients suffered from more severe synovitis and joint damage, and therefore produced more secreted FSTL1 than the male OA patients. However, we did not observe obvious sex difference in the levels of the tissue FSTL1 mRNA expression between the sexes. It is possible that the relatively small tissue sample size overrode the FSTL1 tissue difference between the sexes.

Thus far, biomarkers from cartilage, bone and synovium have been identified in OA patients. The available data have shown that biomarkers from STs perform well in assessing quantitative and dynamic variations in joint remodeling [3]. In this study, we found that FSTL1 expression was strong in the synovial cells and ECs in the STs but relatively low or even absent in the chondrocytes of the articular cartilage in OA patients. These findings suggested that FSTL1 could be a biomarker from synovium. Furthermore, FSTL1 may represent a biomarker of the burden of disease (severity of joint damage) according to the recently proposed BIPED (i.e. burden of disease, investigative, prognostic, efficacy of intervention and diagnostic) classification of OA biomarkers [25]. Our findings also revealed a greater sensitivity of serum FSTL1 levels as an OA biomarker in the female OA patients than in the males. However, we cannot exclude the effects of a relatively small sample size for the male OA patients. Larger cohort studies will be required to better define the potential role of serum FSTL1 as a structural burden biomarker of OA in male patients.

The serum FSTL1 levels correlated with the WOMAC stiffness subscale and they also correlated marginally with the total WOMAC score and WOMAC function subscale in female OA patients. These findings suggested that the increased serum FSTL1 levels were associated with relief of symptoms, including stiffness and function loss, in these patients. Previous reports indicate that FSTL1 ameliorates arthritis by inhibiting the production of proinflammatory mediators [11,12]. Therefore, an explanation is that tissue damage and degradation lead to the release of FSTL1 into SF and serum, and the resulting elevated FSTL1 levels contribute to relief of OA symptoms by inhibiting the production of MMPs and proinflammatory cytokines. However, conflicting data indicate that the increased FSTL1 expression aggravates the effects of arthritis in a mouse model [8,15]. Further investigation is required to assess the exact roles of FSTL1 in OA pathogenesis.

There are several limitations of this study. Firstly, the SF and ST sample size from control trauma patients was relatively small. Also, these samples were not matched with OA samples by age and gender. However, a lower number of control samples is generally inherent to studies of human OA SF and tissue. Secondly, we had limited clinical data for ST and SF because we mostly used banked samples. Furthermore, relatively fewer male OA patients had WOMAC and VAS scores. These factors restricted our assessment to the relationships between FSTL1 expression and the clinical characteristics of these patients.

## Conclusions

In summary, increased FSTL1 expression may be a characteristic of OA patients. FSTL1 is a potential serum biomarker that may reflect the severity of joint damage, and further studies are required to evaluate its potential application for monitoring the course of the disease and the efficacy of therapies in OA patients.

## Abbreviations

ACS: acute coronary syndrome; BMI: body mass index; EC: endothelial cell; ELISA: enzyme-linked immunosorbent assay; ESR: erythrocyte sedimentation rate; FSTL1: follistatin-like protein 1; HCs: healthy control; Hs-CRP: high sensitivity C-reactive protein; IFN: interferon; IgG: immunoglobulin G; IHC: immunohistochemistry; IL: interleukin; JRA: juvenile rheumatoid arthritis; JSN: joint space narrowing; KL: Kellgren and Lawrence; MMPs: matrix metalloproteinases; NSAIDs: nonsteroidal anti-inflammatory drugs; OA: osteoarthritis; RA: rheumatoid arthritis; RF: rheumatoid factor; SF: synovial fluid; ST: synovial tissue; VAS: visual analogue scale; WOMAC score: Western Ontario and McMaster Universities Osteoarthritis score.

## Competing interests

The authors declare that they have no competing interests.

## Authors' contributions

DL conceived and designed the study, interpreted and analyzed data, and drafted and wrote the manuscript. DL, YW, RZ and WF carried out the experiments. YW, NX, WT, RZ and RS completed the acquisition of the clinical samples and data. YW, TD, NX and PZ performed the clinical and laboratory evaluations. LY provided help during the experiment and valuable suggestions. All authors read and approved the final manuscript.

## Supplementary Material

Additional file 1 **Figure 1S**. Distribution of serum FSTL1 concentrations in 48 male and 120 female OA patients. **Table 1S**. Baseline characteristics of 112 female patients with OA of the knee according to serum FSTL1 levels. **Table 2S**. Baseline characteristics of 46 male patients with OA of the knee according to serum FSTL1 levels. Click here for file
