# Supplementary material for: Follistatin-like protein 1: a serum biochemical marker reflecting the severity of joint damage in patients with osteoarthritis
Source: Arthritis Res Ther. 2011 Nov 25;13(6):R193. doi: 10.1186/ar3522 (PMC3334643; doi:10.1186/ar3522)
Supplement: Additional file 1 — Figure 1S. Distribution of serum FSTL1 concentrations in 48 male and 120 female OA patients. Table 1S. Baseline characteristics of 112 female patients with OA of the knee according to serum FSTL1 levels. Table 2S. Baseline characteristics of 46 male patients with OA of the knee according to serum FSTL1 levels. [file ar3522-S1.DOC]

**Additional File 1**

**Figure 1S**

**
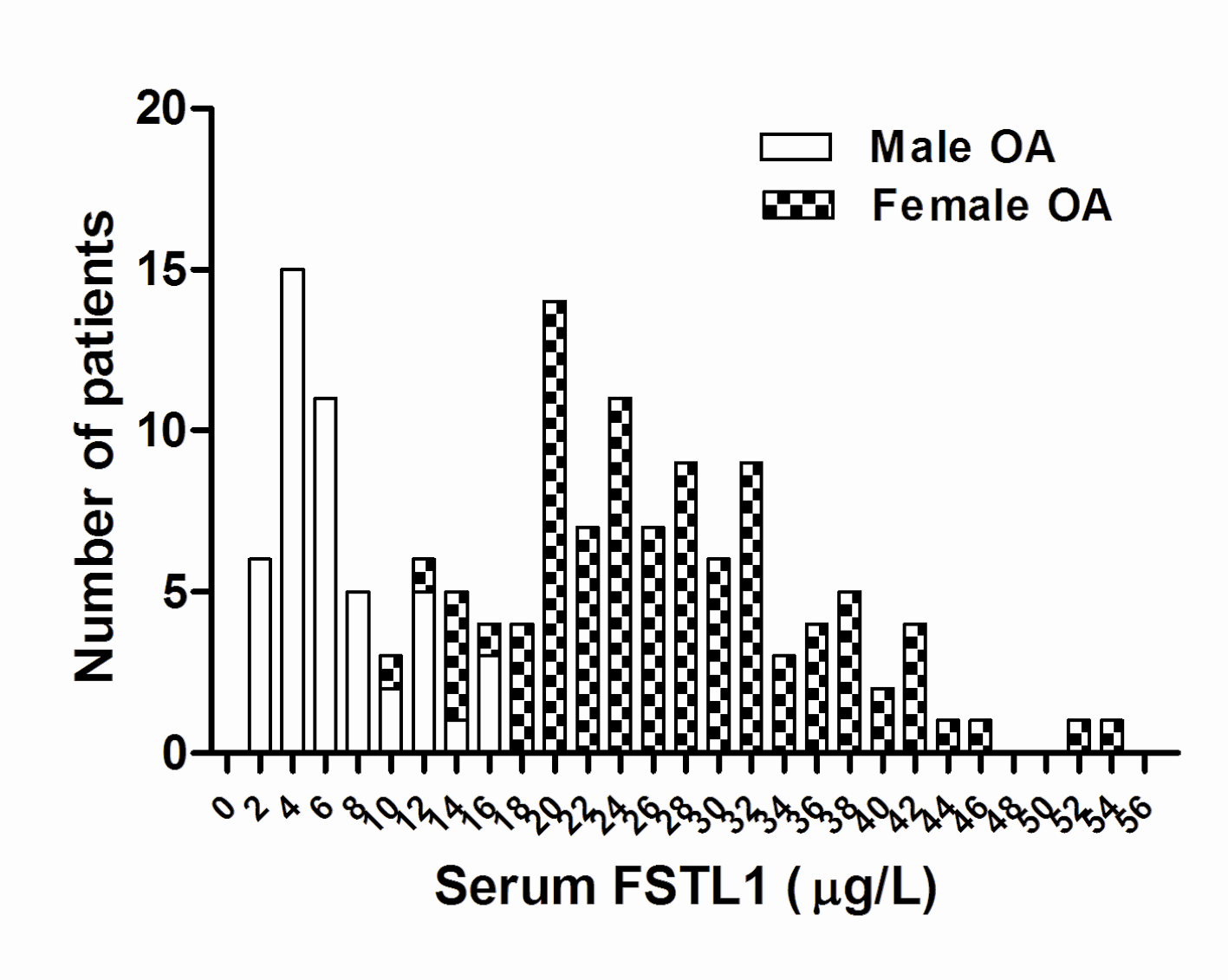
**

**Figure 1S.** Distribution of serum FSTL1 concentrations in 48 male and 120 female OA patients. FSTL1, follistatin-like protein 1; OA, osteoarthritis.

**Table 1S. Baseline characteristics of 112 female patients with OA of the knee according to serum FSTL1 levels.**

|  | Female serum FSTL1 levels (μg/L) | | | |  |  |
| --- | --- | --- | --- | --- | --- | --- |
|  | First quartile | Second quartile | Third quartile | Fourth quartile |  |  |
|  | (1.94-15.09) | (17.42-23.84) | (24.45-30.63) | (30.74-54.20) | *p* | r |
| n | 28 | 28 | 28 | 28 |  |  |
| Age (years) | 62 (52-68) | 66 (57-72) | 62 (56-69) | 62 (54-67) | 0.442 | 0.073 |
| Disease duration (years) | 1 (0.5-3.0) | 1.5 (0.2-6.8) | 1.5 (0.5-3.8) | 1 (0.5-5.0) | 0.629 | 0.046 |
| Height, cm | 160 (156-165) | 158 (156-161) | 158 (158-162) | 158 (155-160) | 0.041 | -0.194 |
| Weight, kg | 65 (60-70) | 63 (55-70) | 65 (60-71) | 60 (55-65) | 0.242 | -0.112 |
| BMI | 25.4 (23.5-27.3) | 25.4 (22.2-28.1) | 25.8 (24.4-27.7) | 23.9 (22.7-26.9) | 0.634 | -0.045 |
| Single/dual knee | 21/7 | 20/8 | 19/9 | 21/7 | 0.693 | 0.038 |
| Hypertension | 9 (32%) | 9 (32%) | 12 (43%) | 5 (18%) | 0.348 | -0.090 |
| Diabetes | 5 (18%) | 2 (7%) | 6 (21%) | 2 (7%) | 0.993 | -0.001 |
| HsCRP (mg/L) | 1.4 (0.7-3.7) | 2.2 (1.2-4.6) | 1.9 (1.1-4.7) | 1.8 (0.8-7.2) | 0.228 | 0.115 |
| ESR^a^ | 13 (7-23) | 11 (8-21) | 10 (6-20) | 11 (5-20) | 0.189 | -0.140 |
| RF | 6.9 (3.3-13.1) | 9.9 (3.9-14.7) | 7.3 (3.4-12.0) | 7.7 (2.7-15.6) | 0.978 | 0.003 |
| Pain (VAS mm)^b^ | 60 (50-70) | 60 (50-80) | 55 (38-80) | 50 (34-63) | 0.085 | -0.186 |
| WOMAC score (all normalized/100)^c^ | |  |  |  |  |  |
| Pain subscale | 50 (48-62) | 61 (50-71) | 50 (46-64) | 61 (51-71) | 0.268 | 0.137 |
| Stiffness subscale | 47 (43-54) | 50 (39-61) | 58 (42-75) | 63 (53-74) | 0.038 | 0.254 |
| Function subscale | 56 (50-68) | 60 (49-69) | 63 (49-78) | 75 (59-90) | 0.083 | 0.213 |
| Total Score | 57 (48-63) | 58 (48-66) | 61 (48-77) | 68 (57-78) | 0.073 | 0.207 |
| KL grade (0-4) | 5/7/13/1/2 | 0/5/8/6/9 | 0/4/10/9/5 | 0/5/2/12/9 | <0.0001 | 0.415 |
| JSN | 3 (11%) | 15 (54%) | 14 (50%) | 21 (75%) | <0.0001 | 0.436 |

Data are n (%) or median (25th-75th percentile). Linear regression was used to evaluate the relationships between the FSTL1 levels and baseline characteristics and to calculate *p* values and the correlation coefficient, r. (Disease duration, hs-CRP, ESR and RF in the female OA patients were not normally distributed and were entered as ln-transformed variables). ^a^Data from ESR were available for 90 patients; ^b^Data from Pain VAS were available for 87 patients; ^c^Data from the total WOMAC score were available for 76 patients, and the pain subscale, stiffness subscale and function subscale were available for 67 patients, respectively. OA, osteoarthritis; FSTL1, follistatin-like protein 1; BMI, body mass index; Hs-CRP, high sensitivity C-reactive protein; ESR, erythrocyte sedimentation rate; RF, rheumatoid factor; VAS, visual analogue scale; JSN, joint space narrowing.

**Table 2S. Baseline characteristics of 46 male patients with OA of the knee according to serum FSTL1 levels.**

|  | Male serum FSTL1 levels (μg/L) | | | |  |  |
| --- | --- | --- | --- | --- | --- | --- |
|  | First quartile | Second quartile | Third quartile | Fourth quartile |  |  |
|  | (1.19-3.79) | (3.81-5.40) | (5.88-8.79) | (9.15-16.88) | *p* | r |
| n | 11 | 12 | 12 | 11 |  |  |
| Age (years) | 54 (45-61) | 60 (54-71) | 63 (52-72) | 70 (64-79) | 0.003 | 0.425 |
| Disease duration (years) | 0.1 (0.1-2.0) | 0.4 (0.2-1.0) | 0.8 (0.3-2.8) | 3.0 (0.2-8) | 0.003 | 0.434 |
| Height, cm | 170 (168-174) | 168 (165-170) | 166 (162-170) | 170 (168-170) | 0.387 | -0.131 |
| Weight, kg | 73 (65-76) | 68 (64-76) | 70 (61-75) | 66 (60-78) | 0.431 | -0.119 |
| BMI | 24.0 (22.5-26.3) | 24.6 (22.7-26.1) | 25.1 (22.6-25.8) | 23.4 (21.1-26.7) | 0.579 | -0.084 |
| Single/dual knee | 10/1 | 12/0 | 10/2 | 8/3 | 0.165 | 0.208 |
| Hypertension | 4 (36%) | 5 (42%) | 5 (42%) | 5 (45%) | 0.568 | 0.086 |
| Diabetes | 2 (18%) | 1 (8%) | 0 (0%) | 3 (27%) | 0.822 | 0.034 |
| HsCRP (mg/L) | 1.2 (0.7-3.3) | 1.4 (0.4-10.7) | 1.5 (0.6-3.6) | 2.3 (0.6-17.1) | 0.068 | 0.272 |
| ESR^a^ | 9 (5-13) | 4 (2-7) | 5 (4-23) | 12 (2-27) | 0.970 | -0.006 |
| RF | 3.5 (0.2-10.2) | 7.2 (4.8-12.1) | 12.9 (6.0-19.5) | 8.8 (7.4-15.4) | 0.021 | 0.340 |
| Pain (VAS mm)^b^ | 60 (40-60) | 40 (20-60) | 50 (30-80) | 60 (40-80) | 0.397 | 0.142 |
| WOMAC score (all normalized/100)^c^ | |  |  |  |  |  |
| Pain subscale | 61 (57-68) | 64 (64-71) | 57 (50-68) | 61 (57-61) | 0.384 | -0.165 |
| Stiffness subscale | 58 (44-60) | 69 (58-86) | 49 (42-79) | 58 (47-58) | 0.647 | -0.087 |
| Function subscale | 63 (54-71) | 81 (56-94) | 65 (57-90) | 60 (52-66) | 0.669 | -0.081 |
| Total Score | 58 (54-75) | 73 (60-83) | 63 (52-78) | 61 (56-64) | 0.371 | -0.164 |
| KL grade (0-4) | 5/4/1/0/1 | 4/2/3/1/2 | 3/1/6/1/1 | 2/2/3/1/3 | 0.061 | 0.279 |
| JSN | 1 (9%) | 3 (25%) | 2 (17%) | 4 (36%) | 0.113 | 0.237 |

Data are n (%) or median (25th-75th percentile). Linear regression was used to evaluate the relationships between FSTL1 levels and the baseline characteristics and to calculate *p* values and the correlation coefficient, r. (Serum FSTL1 levels, disease duration, hs-CRP, ESR and RF in the male OA patients were not normally distributed and were entered as ln-transformed variables). ^a^Data from ESR were available for 37 patients; ^b^Data from Pain VAS were available for 38 patients; ^c^Data from the total WOMAC score were available for 32 patients, and the pain subscale, stiffness subscale and function subscale were available for 30 patients, respectively. OA, osteoarthritis; FSTL1, follistatin-like protein 1; BMI, body mass index; Hs-CRP, high sensitivity C-reactive protein; ESR, erythrocyte sedimentation rate; RF, rheumatoid factor; VAS, visual analogue scale; JSN, joint space narrowing.
